# Identification of Directed Interactions in Kinematic Data during Running

**DOI:** 10.3389/fbioe.2017.00067

**Published:** 2017-10-31

**Authors:** Giovana Y. Nakashima, Theresa H. Nakagawa, Ana F. dos Santos, Fábio V. Serrão, Michel Bessani, Carlos D. Maciel

**Affiliations:** ^1^Federal Institute of Education, Science and Technology of São Paulo, Campus Salto, Salto, Brazil; ^2^Laboratory of Signal Processing (LPS), Electrical Engineering Department (EESC), University of São Paulo, São Carlos, Brazil; ^3^Uninorte, Laureate International Universities, Manaus, Brazil; ^4^Laboratory of Assessment and Intervention in Orthopaedics and Traumatology (LAIOT), Physiotherapy Department, Federal University of São Carlos, São Carlos, Brazil

**Keywords:** partial-directed coherence, Granger causality, kinematics, running, knee

## Abstract

The knowledge of motion dynamics during running activity is crucial to enhance the development of rehabilitation techniques and injury prevention programs. Recent studies investigated the interaction between joints, using several analysis techniques, as cross-correlation, sensitivity analysis, among others. However, the direction of the joints pairing is still not understood. This paper proposes a study of the influence direction pattern in healthy runners by using kinematic data together with partial directed coherence, a frequency approach of Granger causality. The analysis was divided into three anatomical planes, sagittal, frontal, and transverse, and using data from ankle, knee, hip, and trunk segments. Results indicate a predominance of proximal to distal influence during running, reflecting a centralized anatomic source of movements. These findings highlight the necessity of managing proximal joints movements, in addition to motor control and core (trunk and hip) strengthening training to lumbar spine, knee, and ankle injuries prevention and rehabilitation.

## 1 Introduction

Running is a popular activity and about 38 million Americans practice this sport regularly (NSGA, 2011). In recreational and competitive forms, injuries are common and the incidence of musculoskeletal kind ranges from 19.4% to 92.4% (van Gent et al., 2007), with the knee accounting for 50% of all lower extremity problems. Considering that these injuries are associated with altered joints movement (Powers, 2003; Chuter and Janse de Jonge, 2012), a thorough understanding of the complex nature of functional movements is important and could improve prevention, training, and rehabilitation.

Knowledge of joint interaction pattern behavior, mainly the direction of the influence during running, would improve the expertise about the normal and pathological movement, developing musculoskeletal injury treatment (Powers, 2003; Pandy and Andriacchi, 2010). In abnormal motions of the lower extremity, joints could be influenced from the ground and ankle up (i.e., distal to proximal influence) and from the trunk and hip down (i.e., proximal to distal influence) (Powers, 2003, 2010; Chuter and Janse de Jonge, 2012).

A variety of biomechanical studies already investigated the joint interaction and interjoint coordination during functional and athletic activities. Those studies used several analysis techniques, such as cross-correlation and vector coding (Pohl and Buckley, 2008), sensitivity analysis (Nott et al., 2010), coupling angle and continuous relative phase (Chang et al., 2008), and principal component analysis (Tanabe et al., 2014). Due to the fact that the trunk, pelvis, and lower limb kinematic during running are complex, it is not yet fully understood (Pandy and Andriacchi, 2010).

This study is to determine the directed interactions among the three-dimensional (3D) joint kinematic data in healthy recreational athletes during running, by using data channels from ankle, knee, hip, and trunk. The 3D kinematic data were analyzed by anatomical plane, in terms of proximal to distal or distal to proximal influences, by using a frequency-domain approach of Granger causality, named partial directed coherence.

Granger causality concept has been used to determine causal influences among multivariate time series (Ding et al., 2006). In summary, a causal interaction from a process *x*_2_ to a process *x*_1_ is suggested if there is a reduction of prediction error of *x*_1_ when including the past of *x*_2_ (Chicharro, 2011). With a pairwise analysis and assuming that the process is linear, Gaussian and stationary (Chicharro, 2011), the analysis is developed in the time domain from the covariance matrix of the noise terms of the autoregressive (AR) representation of two stochastics processes (Ding et al., 2006). Partial directed coherence (PDC), introduced by Baccalá and Sameshima (2001), is a different approach of Granger causality applied in frequency-domain from Fourier Transform of the multivariate autoregressive (MVAR) coefficients matrix (Graef et al., 2013).

Several studies applied PDC approach. Faes and Nollo (2013) discussed and employed two new measures extending PDC to analyze blocks or groups of a set of time series. Taxidis et al. (2010) assessed interactions between brain regions handling PDC and generalized PDC (gPDC)^1^ in neuronal activity data. gPDC analysis is also implemented in Gürkan et al. (2014) to identify cortical connectivity. Although the extensive use of PDC in neural signals, to the best of the authors’ knowledge, PDC had not been previously employed in joint kinematic data during running. There are some computational issues that concern to the PDC implementation, such as the definition of the best order and the estimation of the coefficients of the MVAR model and the PDC calculation itself. PDC is also a quite new approach when compared to regular methods.

The remainder of this paper is organized as follows. Section 2 introduces the necessary theory to perform pairwise PDC. Section 3 presents the subjects used in this study, the 3D joint kinematic data acquisition procedure, the simulated data used to validate the approach and the developed routines. Section 4 shows the results of the simulated data and the outcomes obtained from the real data in the three anatomical planes. Section 5 discusses the findings with different interpretations present in the literature. Section 6 highlights the main contribution of this study to physical therapy related to lower limb injury and rehabilitation programs.

## 2 Theory

Granger causality uses a linear regression model to establish the idea that if the prediction of a time series *x*_1_ could be improved by including the knowledge of another time series *x*_2_, then one says that *x*_2_ has a causal influence on *x*_1_ (Ding et al., 2006). A pairwise analysis approach is developed in the time domain from the covariance matrix of the noise terms of the AR representation of two stochastics processes (Ding et al., 2006). The *x*_2_ has a causal influence on *x*_1_ if there is a reduction in the variance of the autoregressive prediction error after incorporating *x*_2_ past data when predicting *x*_1_ (Ding et al., 2006).

PDC was introduced by Baccalá and Sameshima (2001) and is one approach of Granger causality to a MVAR model in frequency-domain to infer a direct connection between time series. Consider an n-dimensional random process *X*(*t*) = [*x*_1_(*t*),*x*_2_(*t*), …, *x_n_*(*t*)]*^T^*, where T denotes matrix transposition. The *p*th order MVAR representation of *X*(*t*) is as follows:

(1) $$X\left(t\right)=\sum_{r=1}^{p}\;A_{r}X\left(t-r\right)+E\left(t\right),$$

where *A_r_* are the MVAR estimative coefficient matrices with elements *a_ij_*(*r*) and *E*(*t*) = [*e*_1_(*t*), *e*_2_(*t*), …, *e_n_*(*t*)]^*T*^ is a noise vector. According to Takahashi et al. (2007), by applying the Discrete Time Fourier Transform (DTFT) on the *A_r_* coefficients results in *A*(*f*):

(2) $$A\left(f\right)=\sum_{r=1}^{p}\;A_{r}e^{-\mathrm{ir}2\pi f},$$

where $i=\sqrt{-1}$. The PDC from *x_j_* to *x_i_* is given by

(3) $$\pi_{ij}(f)=\frac{{A^{\prime }}_{ij}(f)}{\sqrt{{{\text{a}}^{\prime }}_{j}{\;}^{H}(f){{\text{a}}^{\prime }}_{j}(f)}},$$

where matrix *A*′ is calculated by:

(4) $$A^{\prime }\left(f\right)=I-A\left(f\right),$$

and, in equation 3, ${\text{a}}_{k}^{\prime }$ is the *k*th column of *A*′, *H* denotes Hermitian matrix and, in equation 4, *I* is identity matrix.

## 3 Materials and Methods

### 3.1 Simulated Data Processing

For validation purpose, a five-channel process was used to generate a data set of 500 observations with 100-time points by channel, similar to Baccalá and Sameshima (2001). The channels are described by the following equations:

(5) $$\begin{array}{l} x_{1}\left(t\right)=0.95\sqrt{2}x_{1}\left(t-1\right)-0.9025x_{1}\left(t-2\right)+e_{1}\left(t\right) \end{array}$$

(6) $$\begin{array}{l} x_{2}\left(t\right)=-0.5x_{1}\left(t-1\right)+e_{2}\left(t\right) \end{array}$$

(7) $$\begin{array}{l} x_{3}\left(t\right)=0.1x_{1}\left(t-2\right)-0.4x_{2}\left(t-2\right)+e_{3}\left(t\right) \end{array}$$

(8) $$\begin{array}{l} x_{4}\left(t\right)=-0.5x_{3}\left(t-1\right)+0.25\sqrt{2}x_{4}\left(t-1\right)+0.25\sqrt{2}x_{5}\left(t-1\right)+e_{4}\left(t\right) \end{array}$$

(9) $$\begin{array}{l} x_{5}\left(t\right)=-0.25\sqrt{2}x_{4}\left(t-1\right)+0.25\sqrt{2}x_{5}\left(t-1\right)+e_{5}\left(t\right) \end{array}$$

where *e_i_*(*t*) represents white noise processes with zero means and variances equal to one. The generated data will represent the following causal relations among the channels (the arrows point influence direction):

- *x*_1_ →*x*_2_
- *x*_1_ →*x*_3_ and *x*_2_ →*x*_3_
- *x*_3_ →*x*_4_ and *x*_5_ →*x*_4_
- *x*_4_ →*x*_5_.

The Python Nitime (http://nipy.org/nitime/) library was used to estimate the MVAR coefficients, with order two to calculate the PDC values.

### 3.2 Subjects

Twenty-nine healthy recreational runners participated in this study (average (SD); age 27.67 (5.43) years, mass 72.05 (13.61) kg, body mass index 23.74 (2.92) kg/m^2^, height 1.73 (0.09) m, average running distance of 35.70 (18.25) km/week, and running experience 4.13 (4.02) years). All the participants met the following criteria: they were rearfoot strikers, familiar with treadmill running and ran a minimum of 20 km/week at least 3 months prior to study enrollment.

All the subjects were evaluated by a licensed physical therapist. The exclusion criteria were the presence of bone, joint, or ligament injury occurred in the least 3 months prior the assessment, lower limb surgery, the presence of pain in the ankle, knee, hip, or trunk while running or wearing orthotics that could interfere with their running pattern. The testing protocol was approved by the Federal University of São Carlos Ethics Committee for Human Investigations, and the subjects signed a written informed consent form to participate in this study.

### 3.3 Data Acquisition Procedure

The acquisition session started with a 5-min warm-up on a treadmill (model LX 160 GIII, Movement, Manaus, Brazil) at 1.38 m/s. The subjects were then instructed to start running at a comfortable speed, determined by the volunteer and adjusted by the assessor for 2 min. A neutral running shoe (Asics Gel-Equation 5, ASICS, Kobe, Japan) was provided for all runners. Each stored signal had a total length of 1 min and 30 s and was acquired without informing subjects about the exact moment of sampling nor the variables were studied.

**Figure 1:**
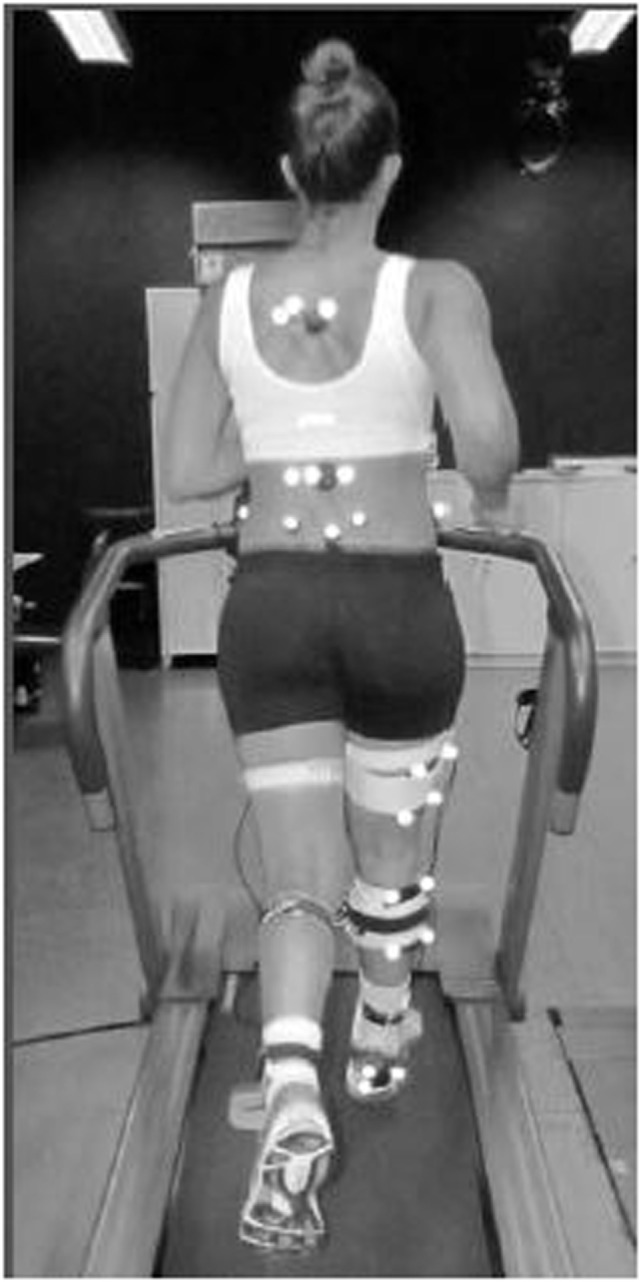
Subject running on a treadmill with the reflective markers (little white circles) in the trunk, hip, knee, and ankle during a kinematic data collection trial.

The kinematic data^2^ of the dominant lower limb (5 left, 26 right) and trunk were recorded at 240 Hz during running with a six-camera Qualisys motion analysis system (Qualisys Inc., Gothenburg, Sweden). Twenty reflective markers located on anatomical landmarks and five cluster tracking markers were placed on each subject (Figure 1). The reliability of the analyzed variables was Intraclass Correlation Coefficient (ICC) 0.73–0.91, with 95% of confidence interval (CI).

The Cardan angles were calculated using the joint coordinate system definitions recommended by the International Society of Biomechanics (Wu et al., 2002) relative to the static standing trial using the Visual 3D software (C-Motion Inc., Rockville, MD, USA). The kinematic data were filtered with the Visual 3D software using a fourth order, zero lag, low-pass Butterworth filter at 12 Hz. For each plane (X—sagittal, Y—frontal, and Z—transverse), four joints were collected: ankle, knee, hip, and trunk.

### 3.4 Real Data Processing

As a preprocessing procedure, each kinematic data channel was normalized by their root mean square (RMS) value. For each subject, MVAR estimative coefficient matrix was computed using the Nitime library, covering all channels, following equation 1. The MVAR order was evaluated by using the Bayesian Information Criterion (BIC) for each order in a range from one to one thousand, and the order with smallest BIC value was chosen. The pairwise analysis was performed in each plan, and PDC values were calculated according to equation 3. From the values of PDC over the frequency range of an interaction, the maximum represented the influence between the two channels (Baccalá and Sameshima, 2001; Faes and Nollo, 2013).

A set of the 29 maximum PDC values was generated for the twelve possible interactions between the joints. The Shapiro–Wilk test was employed in order to assess the presumption of normality (Razali and Wah, 2011). Mean values (Gürkan et al., 2014) were used in order to verify the hypothesis that the proximal–distal and distal–proximal interactions were equal. The t-test was applied to the groups in which there was an indication that the data follow a normal distribution, and permutation test (Ernst, 2004), with *N* = 10,000 permutations, for all groups. Both tests used adjusted p-values determined by Bonferroni correction (Goeman and Solari, 2014) with α = 0.025 and were shown in three tables, each one for one plane. Also, mean values were plotted in three distinct graphs.

All computational routines were developed in Python 2.7.4 (Python Software Foundation, USA), and executed on an Intel Core i5 (Intel Corporation, USA) CPU at 1.70 GHz, 4 GB RAM and Ubuntu 13.04 operating system (Canonical Ltd., UK).

## 4 Results

### 4.1 Simulated Data Processing

**Figure 2:**
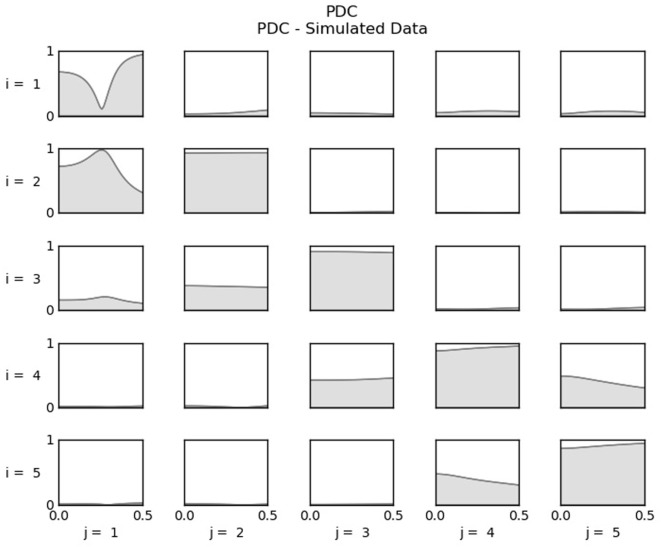
PDC values computed from the simulated data. For each cell, the influence is from *j*-channel to *i*-channel. Thus, in the second line, the first plot shows the influence from channel one (*j* = 1) to channel two (*i* = 2).

The synthetic data generated following the equations presented in Section 3.1 produced the result shown in Figure 2. The PDC values for influences from *j* (column) channel to *i* (line) channel are presented for the entire frequency range as the gray shaded areas. A cell in the third line and second column shows the influence from channel two to channel three.

From Figure 2 is possible to infer the influence among the five channels, *x*_1_ receives no influences; *x*_2_ is influenced only by *x*_1_; *x*_3_ is influenced by *x*_1_ and *x*_3_; *x*_4_ receives influence from *x*_3_ and *x*_5_; and *x*_5_ is influenced by *x*_4_. All other influences are depicted as gray areas equal or very near to zero. The result obtained is in agreement with the equations used to generate the synthetic data in Section 3.1 and indicates the computational routine is working properly.

### 4.2 Real Data Processing

**Figure 3:**
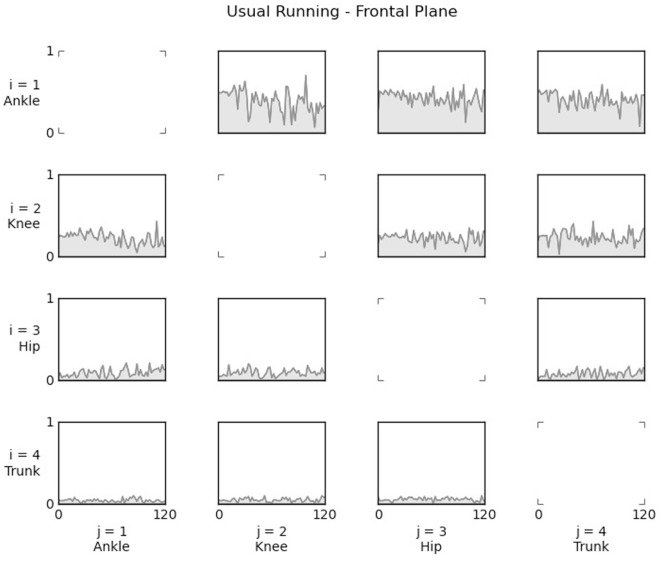
PDC values computed from 3D kinematic data, in the sagittal plane, collected during usual running from one subject. For each cell, the influence is from *j*-channel to *i*-channel. Thus, in the second line, the first plot shows the influence from the ankle (*j* = 1) to the knee (*i* = 2).

The PDC values obtained for each pair of 3D joint kinematic data channels were analyzed for each of the three planes separately. Ankle, knee, hip, and trunk kinematic joints interactions were properly evaluated in sagittal, frontal, and transverse planes. For illustration purposes, the Figure 3 presents a four by four matrix, where in each cell was plotted PDC values for the entire frequency range between pairs of channels from a single subject. As in simulated data, the influences are from *j* (column) channel to *i* (line) channel. Thus, the first graphic in line two shows the interaction from the ankle (channel one) to the knee (channel two).

**Figure 4:**
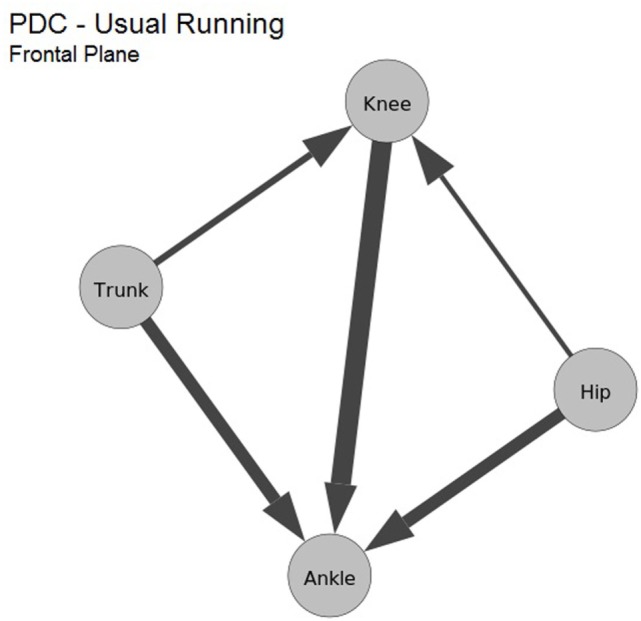
Causal influence in the kinematic data during the running of one subject, in the frontal plane. The nodes are the 3D kinematic joints. Each edge represents the maximum PDC computed between the joints.

Figure 4 shows the influence patterns in the frontal plane for a single evaluated subject. The joints are represented as nodes in the graph, and the maximum computed PDC values for each pair of data channels are drawn as directed edges, where their direction is the same as the influence and thickness reflect the maximum PDC value. Thus, a wider edge indicates a higher influence pattern between two joints, e.g., the influence from hip to ankle has a higher value than the interaction from hip to knee.

Additionally, the average and SD of maximum PDC values were calculated for all subjects and each influence plane. The influence pattern analysis consists in using a hypothesis test (t-test and permutation test with a significance level defined by Bonferroni correction of 0.025) to compare the obtained average values and infer how the influence happens, from distal to proximal or from proximal to distal.

Distal and proximal are anatomical terms of location; the distal term designates a location that is distant from the body center or some anatomical point of reference, and the proximal term is the location closer from them. Accordingly, in the ankle–knee pair, distal–proximal influence describes the interaction from ankle to knee, and proximal–distal, from knee to ankle. The paired t-test and permutation tested the null hypothesis that average value from distal to proximal influence was equal to proximal to distal average value. The t-test was applied only when there was an indication of normality.

**Figure 5:**
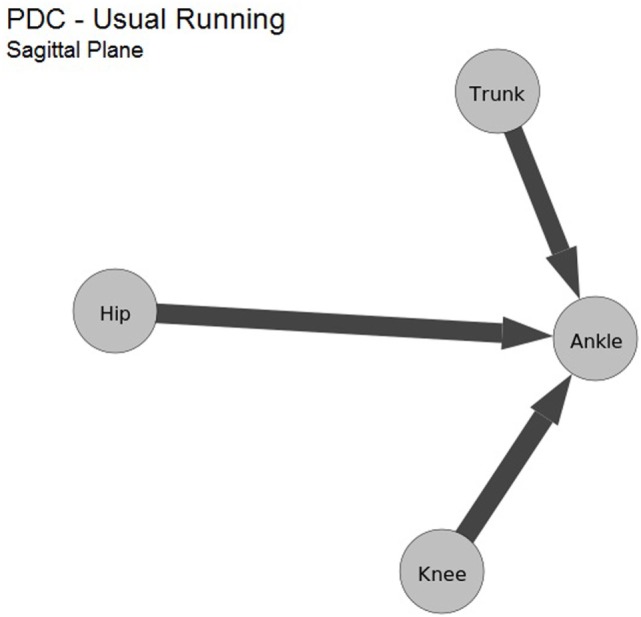
Average values computed from maximum PDC values of the sagittal plane kinematic data during running. As in Figure 4, the nodes are the kinematic data.

In the sagittal plane, a greater proximal to distal influences was found in comparison to distal to proximal in three of six combination pairs of joints (ankle–knee, ankle–hip, and trunk–ankle), the other three combination pairs resulted in a similar influence, as shown in Table 1. In Figure 5, these influences are represented in a directed graph of the sagittal plane. By inspecting the graph, it is evident that the ankle is the joint that suffers the greater influence in the sagittal plane.

**Table 1:** Average values (SD) computed from causal influence in the sagittal plane kinematic data during running.

Pair	Distal–prox	Proximal–distal	t-test	Permutation test	Influence
Ankle–knee	0.24 (0.14)	0.72 (0.13)	–	0.000	Prox–distal
Ankle–hip	0.21 (0.10)	0.69 (0.16)	–	0.000	Prox–distal
Knee–hip	0.24 (0.12)	0.26 (0.13)	–	0.593	∼
Trunk–hip	0.30 (0.27)	0.23 (0.12)	–	0.200	∼
Trunk–knee	0.28 (0.26)	0.23 (0.15)	–	0.327	∼
Trunk–ankle	0.29 (0.27)	0.68 (0.13)	–	0.000	Prox–distal

**Figure 6:**
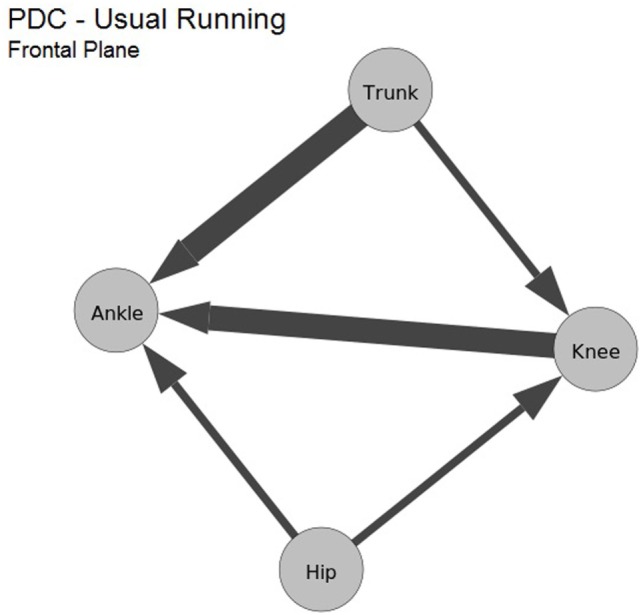
Average values computed from maximum PDC values of the frontal plane kinematic data during running. Also, nodes are the kinematic data.

Table 2 presents the average values computed for the frontal plane kinematics. Proximal to distal influence was greater in five (ankle–knee, ankle–hip, knee–hip, trunk–knee, and trunk–ankle) of six pairs of joints, the other pair resulted in a similar influence. Figure 6 shows the graph representation of those influences. Analogous to the findings for the sagittal plane, the ankle is again the joint that suffers most of the other joints influence, followed by the knee.

**Table 2:** Average values (SD) calculated from causal influence in the frontal plane kinematic data during running.

Pair	Distal–prox	Proximal–distal	t-test	Permutation test	Influence
Ankle–knee	0.45 (0.17)	0.75 (0.15)	–	0.000	Prox–distal
Ankle–hip	0.35 (0.13)	0.73 (0.17)	–	0.000	Prox–distal
Knee–hip	0.35 (0.11)	0.48 (0.18)	0.002	0.002	Prox–distal
Trunk–hip	0.36 (0.15)	0.36 (0.12)	0.890	0.885	∼
Trunk–knee	0.33 (0.12)	0.47 (0.18)	0.003	0.001	Prox–distal
Trunk–ankle	0.32 (0.12)	0.77 (0.12)	0.000	0.000	Prox–distal

**Figure 7:**
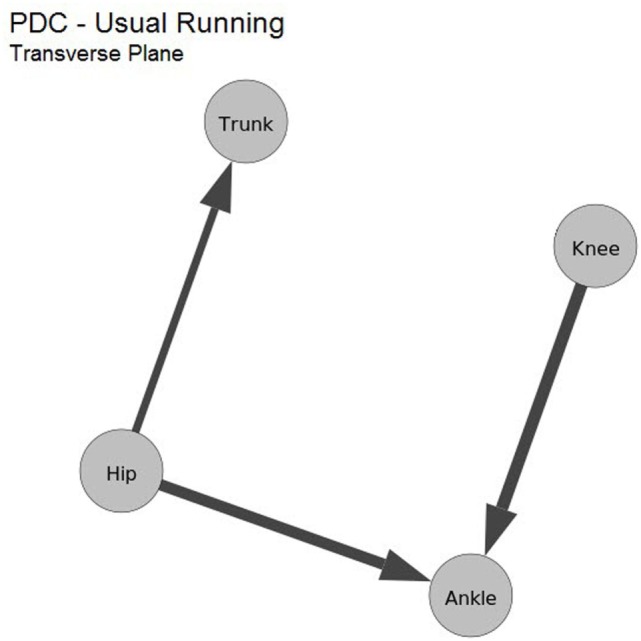
Average values computed from maximum PDC values of the transverse plane kinematic data during running. Also, nodes are the kinematic data.

In the transverse plane, of six pairs of joints, two of them presented more proximal to distal influence (ankle–knee and ankle–hip), and one pair presented distal to proximal influence (trunk–hip), the other three resulted in similar influence for both directions. The obtained values are shown in Table 3 and its graph representation is presented in Figure 7. Differently from the sagittal and frontal planes, for the transverse plane, an influence distal to proximal was indicated.

**Table 3:** Average values (SD) computed from causal influence in the transverse plane kinematic data during running.

Pair	Distal–prox	Proximal–distal	t-test	Permutation test	Influence
Ankle–knee	0.41 (0.13)	0.58 (0.15)	0.001	0.000	Prox–distal
Ankle–hip	0.34 (0.14)	0.56 (0.16)	0.000	0.000	Prox–distal
Knee–hip	0.36 (0.14)	0.41 (0.13)	0.110	0.131	∼
Trunk–hip	0.47 (0.15)	0.35 (0.12)	0.001	0.001	Distal–prox
Trunk–knee	0.46 (0.16)	0.38 (0.14)	0.040	0.045	∼
Trunk–ankle	0.45 (0.17)	0.54 (0.17)	–	0.063	∼

## 5 Discussion

This study was to determine the patterns of directional influence among ankle, knee, hip, and trunk, in the sagittal, frontal, and transverse planes separately in kinematic data from healthy runners. Such pattern is of interest for physical therapists, as it can be used to distinguish between normal and pathological running movement, thus aiding runners training, injury prevention programs, and rehabilitation. The patterns were investigated using the PDC model, a frequency-domain approach of Granger causality.

In the PDC calculation, the first step was the MVAR coefficients estimation, which covered all data channels acquired simultaneously. The model order is necessary to estimate the AR coefficients, and BIC was chosen to evaluate the best order in a range from one to one thousand. Most of the processing time, individually, about 2 h 17 m 56 s was due to the model order determination; input and normalization data, 3 m 40 s, PDC processing, 22 s and output data and graphs, 16 s.

In a case study with the entire group of subjects, the final results derived from mean and maximum values were identical in sagittal and frontal planes; in transverse plane, there was only one different finding between trunk and ankle. From this conclusion and considering that Baccalá and Sameshima (2001) and Faes and Nollo (2013) employed the maximum value computed, this value was used as the descriptor of the interaction between two joints.

The sagittal plane results (Table 1; Figure 5) reveal that, in this plane, the ankle was strongly influenced by the trunk, hip, and knee, in agreement with the study Saha et al. (2008), which reported that healthy subjects that adopted forward trunk lean had presented greater hip, knee, and ankle flexion angles during walking. It was hypothesized that the increased trunk and hip flexion (proximal joints) could lead to an anterior shift of the center of mass. As an adaptation to produce a posterior shift of the trunk and maintain balance, the subjects performed greater knee flexion resulting in a greater ankle dorsiflexion (distal joint). These kinematic adaptations could lead to the crouching posture characterized by greater hip, knee, and ankle flexion angles.

The results cited were the first evidence of a greater proximal influence of the trunk, hip, and knee movement over the most distal joint (ankle) during running, which corroborate with our results, a greater proximal to distal influence from the trunk, hip, and knee to the ankle. Giving that the ankle is the first joint to contact the floor, helping to absorb the impact load during running; the physical therapist might aim to instruct the patient to manage the trunk, hip, and knee kinematics to achieve an optimal ankle angle in the sagittal plane during running.

In the frontal plane, the experiments pointed to a higher proximal to distal influence between ankle–knee, ankle–hip, knee–hip, trunk–knee, and trunk–ankle, shown by Table 2 and Figure 6. As in sagittal plane, the ankle receives the bigger influences from the proximal joints (trunk, hip, and knee). In this plane, the knee appeared as the destination of the interactions from trunk and hip. In fact, for the frontal plane, it has been previously reported that greater ipsilateral trunk angle and knee abduction occurred in combination during jumping in female athletes with non-contact anterior cruciate ligament injury (Hewett et al., 2009), single leg squat (Nakagawa et al., 2012), and running (Noehren et al., 2012) in subjects with patellofemoral pain.

This probably occurs because the trunk comprises more than half of the body’s mass. Thus, ipsilateral trunk motion increases the ground reaction force passing lateral to the knee and, consequently, the knee abduction load (Hewett et al., 2009). Additionally, peak ipsilateral trunk lean was found to be positively associated with knee abduction in healthy subjects during single leg squat (Nakagawa et al., 2015). Given that the greater hip (proximal) influence on the knee (distal), it is important to note that the knee valgus may be a result of hip adduction in the frontal plane (Powers, 2003). A higher knee valgus may increase the dynamic quadriceps angle, with larger lateral vector on the patella, which potentially increase the stress on the lateral compartment of the patellofemoral joint (Powers, 2003).

The results corroborate with previous studies and add the information that the coupling between joints in the frontal plane during running is a result of proximal joints kinematic influencing on the distal joints. This new information is especially important when we take into consideration that the joint movements in the frontal plane are involved in several knee injuries. In light of this, the clinicians should consider training the control of the trunk and hip movement during prevention program and rehabilitation to avoid abnormal and risk movement patterns at the knee.

In the transverse plane, a significantly greater proximal to distal influence between ankle–knee and ankle–hip is shown by Table 3 and Figure 7. Distal to proximal interaction was suggested between trunk–hip. In this plane, it seems that the four kinematic joints were divided into three segments: 1-trunk, 2-hip, and 3-knee and ankle, and that segment two (hip) influences both ankle (proximal–distal) and trunk (distal–proximal). The lower limb joints coupling in the transverse plane have been extensively studied (Chang et al., 2008; Pohl and Buckley, 2008), as excessive or prolonged foot pronation has been linked to the development of numerous overuse injuries affecting the lower limb (Tiberio, 1987; Beckett et al., 1992; Kaufman et al., 1999).

Tiberio (1987) and Chuter and Janse de Jonge (2012) suggested a model where foot motion has more effect on tibia, femur, and hip in the transverse plane. Furthermore, research evidence supports the presence of a dynamic coupling mechanism between lower limb segments (Chang et al., 2008; Pohl and Buckley, 2008), the direction of the coupling is inconclusive. Since PDC analysis gives the direction interaction information between joints data, it has the potential to fill this gap of knowledge.

Notably, a proximal to distal influence from the hip and knee to the ankle were found, an indication contrary to what had been previously proposed that the influence among these joints is greater in the distal to proximal direction. Corroborating with our results that hip influences ankle and trunk, the hip muscle weakness and altered kinematics has been implicated in the lower limb and lumbar spine injuries (Dananberg, 1993; Barnes et al., 2008). In fact, in the transverse plane, the hip joint presents a great range of motion and large muscles (especially the gluteus maximus), as compared to the other evaluated joints explaining its potential to influence the distal and proximal joints. Clinically, these results support hip muscle strengthening and kinematic training to manage and prevent lower limb and trunk injuries.

Most influences are greater in the proximal to distal direction, when considering all the planes, indicating a centralized anatomic source of movements. Overall, our results highlight the importance of managing proximal joints kinematic, in addition to core (trunk and hip) strengthening and motor control training, in order to prevent and rehabilitate lumbar spine, knee, and ankle injuries. Since joints can have an interplane influence between each other and muscular activation may cause kinematic adaptations, future studies should consider analyzing PDC approach in the interplane kinematic data and between electromyographic and kinematic data, respectively.

## 6 Conclusion

In physical therapy, the biomechanical knowledge of running is of great interest since that could improve rehabilitation programs and prevent injuries. In order to provide information of directed pattern influences among 3D kinematic data during running experiments, this work implemented and analyzed results from PDC concept, a frequency approach of Granger causality, which indicates directional influence.

From kinematic data collection during running, the movement of the ankle, knee, hip, and trunk joints were analyzed, in all three anatomical planes, sagittal, frontal, and transverse. PDC values were computed for each plane and pair of segments. In the sagittal plane, the ankle was more strongly influenced by the trunk, hip, and knee; whereas, in the frontal plane, the knee appeared as the destination of the interactions from trunk and hip. Finally, the hip influenced both ankle (proximal–distal) and trunk (distal–proximal) and knee influenced ankle (proximal–distal) in the transverse plane.

Overall, it was demonstrated a greater proximal to distal influences among the four joints, with the ankle being more influenced by knee, hip, and trunk in all three planes. This prevalence of proximal to distal influences indicates that physical therapist should consider including the motor control training of the proximal joints kinematic in the prevention injury programs and rehabilitation of lower limb and trunk conditions.

Future studies are necessary to further investigation of the directed influence of interplane kinematic data, electromyographic data, between stance and swing phases, during distinct running techniques. Besides, it would be interesting to investigate the effect of different running technique and musculoskeletal injuries on the directed influence of kinematic data between joints.

Moreover, the risk of injury could be indicated by the comparison between results obtained from the kinematics of healthy and injured volunteers, by contrasting the direction of influence between joints in runners with and without lower limb injury, before and after rehabilitation, with different running technique, and between stance and balance phase of running. An automated decision support system could be developed by using the findings from such type of studies.

## Ethics Statement

The testing protocol was approved by the Federal University of São Carlos Ethics Committee for Human Investigations, and the subjects signed a written informed consent form to participate in this study.

## Author Contributions

GN, TN, and CM formulated the presented idea. TN and AS carried out the experiment. GN implemented the computer routines with aid from MB. GN, TN, AS, and MB wrote the manuscript with support from CM and FS. TN and AS contributed to the analysis of the results. All authors discussed the results and contributed to the final manuscript.

## Conflict of Interest Statement

The authors declare that the research was conducted in the absence of any commercial or financial relationships that could be construed as a potential conflict of interest.

## Footnotes

^1^gPDC is used in time series with widely different variances.

^2^Available in https://github.com/lablps/FBB2017.
